# Remarks on Dr. Scudamore and Mr. Bampfield, Respecting Colchicum

**Published:** 1822-03

**Authors:** 


### THERAPEIA.

### Art. VI.—*Remarks on* Dr. Scudamore *and* Mr. Bampfield, *respecting Colchicum.* By Dr. Kinglake.

I HAVE to request a page in the next Medical Journal, if possible, to offer a few remarks on a controversy, in which I am mentioned, that has arisen between Dr. Scudamore and Mr. Bampfield, concerning the comparative efficacy of different preparations of colchicum in relieving and curing *gout*. The vinous, aqueous, and acetous, impregnations of that vegetable substance have been severally employed, and are held to possess various degrees of remedial power. The contest on this diversity of preparation seems to rest on a very finely-spun distinction; so fine, indeed, as hardly to amount to a difference. The combatants, on the alleged ground of disputation, may adjust the imaginary disagreement, without losing an iota, either of the correctness which they have respectively assumed, or of the real value of the medicine in question. Before warmly debating which of the enumerated preparations should be preferred, the disputants would have done right cautiously to have settled the doubtful point, whether or not either of them deserves to be regarded as an efficient medicine in gouty disease.

Colchicum appears to possess a narcotic power, nearly analogous to that of hyoscyamus, which is exerted, in both these agents, by torpifying or deadening, as it were, the sensorial property of life. The relief resulting from this influence consists in a temporary suspension of pain, or, rather, incapacity for feeling it. The *methodus medendi*, or agency by which this effect is produced, is similar to that which arises from the use of opium, and which is very directly and uniformly afforded by the compound powder of ipecacuanha, in which opium is well known to be an active ingredient.

My experience of colchicum has been sufficient to convince me that it has no power over painful or distempered sensibility, which might not be as efficiently, and more infallibly, obtained from an appropriate employ of opium; and, still more commodiously, from either the extract or tincture of hyoscyamus. These quieting remedies act by inducing changes in the evolution and distribution of the nervous or sensorial influence that render the affected part susceptible of painful excitement. The effect is, happily, but temporary: if it were more impressive and durable on the energies of life, it would, at least, endanger partial paralysis, if not an entire extinction of vital power.

It has occurred to me to see a deplorable case of gouty discase, in which the free use of colchicum appeared to have induced distressing torpor, general decrepitude, and oppressive congestions of the brain and liver, which, after the lapse of some months' irremediable suffering, terminated in death. If colchicum should become generally employed for the relief either of gout or any other painful affection, it is probable that the temporary alleviation, which it may effect, will be at the expence of incurring various dangerous and destructive maladies.

Inflammatory gout is not within the reach of narcotic influence. It may so affect the sentient conditions of life as greatly to lessen the natural susceptibility for pain ; but this is most likely affected by loading and oppressing the brain, and thereby occasioning an hazardous depression of nervous energy. Such an effect is a more threatening state of disease than the gouty pain that was intended to be subdued. The high morbid action constituting gouty inflammation, requires local applications and general depletion. It cannot be either safely or effectually cured, but by abstracting every kind of hurtful excitement. The sanguiferous system, in gouty disease, stands in need of evacuations, that the circulating fluids may not be unduly detained or obstructed, but be equally distributed over the frame. A gorged liver, an undigesting stomach, and constipated bowels, will induce and sustain gouty ailment, in defiance of colchicum or any other narcotic.

Evaporating applications to the affected part, whether cold or tepid, very directly, salutarily, and effectually, relieve the painful inflammation of gout, and prevent the unabated torture from being injuriously propagated over the system. Appropriate evacuations, and topical cold over the part suffering from gouty inflammation, afford, in almost every instance, early, progressive, and complete relief, with perfect safety and benefit to the general health. The decided advantages of this treatment are sufficiently attested to warrant a full persuasion of its being far preferable to any effect that could result from the superior and insidious agency of either colchicum or any other kindred narcotic.

Mr. Bampfield asserts, in reference to myself, that topical cold in gouty inflammation is a proposition that "has been most severely criticised ;" and then he playfully founds, on the presumed inadmissibility of that remedy, a syllogism, to prove that Dr. Scudamore's evaporating lotion, in that complaint, must be injurious. This sage device runs as follows :—"Cold applications in gout are frequently injurious ; evaporation produces intense cold ; therefore, evaporating lotions must be injurious."

The popular, as well as medical, notion of cooling applications being hurtful in gout, has arisen from the preposterous opinion that gouty pain, contrary to what happens in all other diseases, is remedial, and ought rather to be encouraged than subdued! The boasted efficacy of colchicum in gouty disease acts by allaying pain; yet it is, inconsistently, said to be salutary. Mr. Bampfield is, therefore, requested to accept of the subjoined syllogistic retaliation, in answer to the logical sophistry, which he has adduced against the use of topical cold in gouty inflammation.

*Syllogism.*—Gouty pain is held to be salutary;
Colchicum removes gouty pain;
Therefore, in gouty pain, colchicum cannot be salutary.

*Taunton; Feb. 3d,* 1822.

